# Interleukin-1β inhibitors for the management of acute gout flares: a systematic literature review

**DOI:** 10.1186/s13075-023-03098-4

**Published:** 2023-07-25

**Authors:** Naomi Schlesinger, Michael H. Pillinger, Lee S. Simon, Peter E. Lipsky

**Affiliations:** 1 Division of the Rheumatology at the Spencer Fox Eccles School of Medicine, University of Utah, Harold J, Ardella T, and Helen T Stevenson Presidential Endowed Chair of Rheumatology, Salt Lake City, UT 84132 USA; 2 The Division of Rheumatology, NYU Grossman School of Medicine, New York, USA; 3 SDG, LLC, Cambridge, MA USA; 4 AMPEL BioSolutions, LLC, Charlottesville, VA USA

**Keywords:** Gout flare, Interleukin-1β, Randomised controlled trials, Canakinumab, Rilonacept, Anakinra

## Abstract

**Objectives:**

The objective of this systematic review was to assess the effects of interleukin-1β (IL-1β) inhibitors on gout flares.

**Methods:**

Studies published between 2011 and 2022 that evaluated the effects of IL-1β inhibitors in adult patients experiencing gout flares were eligible for inclusion. Outcomes including pain, frequency and intensity of gout flares, inflammation, and safety were assessed. Five electronic databases (Pubmed/Medline, Embase, Biosis/Ovid, Web of Science and Cochrane Library) were searched. Two independent reviewers performed study screening, data extraction and risk of bias assessments (Cochrane Risk of Bias Tool 2 for randomised controlled trials [RCTs] and Downs and Black for non-RCTs). Data are reported as a narrative synthesis.

**Results:**

Fourteen studies (10 RCTs) met the inclusion criteria, with canakinumab, anakinra, and rilonacept being the three included IL-1β inhibitors. A total of 4367 patients with a history of gout were included from the 14 studies (*N* = 3446, RCTs; *N* = 159, retrospective studies [with a history of gout]; *N* = 762, post hoc analysis [with a history of gout]). In the RCTs, canakinumab and rilonacept were reported to have a better response compared to an active comparator for resolving pain, while anakinra appeared to be not inferior to an active comparator for resolving pain. Furthermore, canakinumab and rilonacept reduced the frequency of gout flares compared to the comparators. All three medications were mostly well-tolerated compared to their comparators.

**Conclusion:**

IL-1β inhibitors may be a beneficial and safe medication for patients experiencing gout flares for whom current standard therapies are unsuitable.

**Review protocol registration:**

PROSPERO ID: CRD42021267670.

**Supplementary Information:**

The online version contains supplementary material available at 10.1186/s13075-023-03098-4.

## Introduction

Gout is a common form of inflammatory arthritis [1, 2] caused by the deposition of monosodium urate (MSU) crystals, which form in the setting of elevated serum urate concentrations (hyperuricemia). [2] Gout initially presents as intermittent acute flares, typically affecting the lower extremities, especially the first metatarsophalangeal joint of the foot. [2, 3] Gout may transition to a chronic state, including polyarticular flares, symptoms between flares, and granuloma-like MSU crystal deposition in soft tissues and/or joints (tophi). [4].

Non-steroidal anti-inflammatory drugs (NSAIDs), colchicine, and steroids are first-line treatment options to control inflammation and pain associated with gout flares, and to prevent flares during the initiation of urate-lowering therapy, which has a high flare risk. [5–7] However, many patients do not respond to/tolerate these therapies, or have an absolute or relative contraindication to their use. [8].

Interleukin-1β (IL-1β) plays a pivotal role in mediating gouty inflammation, and its blockade has demonstrated efficacy in combating gout-related pain and inflammation. [6] In patients who do not respond to standard therapies, guidelines recommend considering IL-1β inhibitors as a treatment for gout flares. [5, 7, 9–11] To date, only one systematic review, published in 2014, has focused on using IL-1β inhibitors to treat gout flares. [12] Therefore, an updated systematic literature review focusing on IL-1β inhibitors is warranted to update the available evidence for their use in treating gout flares.

The objective of this systematic review was to evaluate the accumulated evidence on the effects of IL-1β inhibitors on gout flares.

## Methods

### Registration and protocol

This systematic review is reported according to the Preferred Reporting Items for Systematic Reviews and Meta-Analyses (PRISMA) 2020 guidelines. [13] The protocol was registered with the National Institute for Health Research, International Prospective Register of Systematic Reviews (PROSPERO; ID: CRD42021267670) prior to the initiation of this systematic review.

### Eligibility criteria

The population, intervention, comparison, outcome, and study design (PICOS) framework was used to consider the eligibility of articles for this review.

#### Participants

Eligible participants included male and female adults aged ≥ 18 years experiencing gout flares. Participants with flares caused by other rheumatic diseases such as rheumatoid arthritis and ankylosing spondylitis were excluded.

#### Interventions/exposures

Any intervention using IL-1β inhibitors to treat gout flares was eligible for inclusion. No restriction was applied for intervention duration.

#### Comparators

Comparators eligible for inclusion included recommended treatments for gout flares (i.e., NSAIDs, colchicine, steroids) and/or placebo.

#### Outcomes

The primary outcome measure was pain and inflammatory features associated with gout flares. Outcome measures included pain measurements; number, severity, and duration of gout flares; global response to treatment; and measurements of synovitis. Other outcomes included safety, quality of life (QoL), biomarkers, assessment of clinical signs, and medication use.

#### Studies

Eligible studies were randomised controlled trials (RCTs), quasi-RCTs, non-RCTs and observational studies (both prospective and retrospective) that used IL-1β inhibitors to treat gout flares. Animal studies, in vitro studies, case reports, review articles, letters to the editor and protocols were excluded. Studies not reported in English were also excluded. No sample size limitations were applied.

### Information sources

Electronic databases searched included PubMed/Medline, Embase, Biosis/Ovid, Web of Science, and Cochrane Library. The search was restricted to publication years 2011‒2022. The search was last performed on 23^rd^ November 2022.

### Search strategy

Search strategies were developed and adapted for each electronic database. Keywords including ‘gout flares’, ‘IL-1-beta’, ‘canakinumab’, ‘rilonacept’ and ‘anakinra’ were used to search for relevant articles. The search strategy is provided in the Supplementary Methods.

### Study records

#### Data management

Retrieved studies were imported into the Covidence software (Covidence systematic review software, Veritas Health Innovation, Melbourne, Australia. Available at www.covidence.org) for screening. The number of all included and excluded records, including reasons for exclusion, where applicable, were detailed in a PRISMA flow diagram.

#### Selection process

Two independent reviewers screened the records at the title and abstract level and then at the full-text level based on pre-defined eligibility criteria. Any conflicts arising during the screening were resolved by consensus or through discussion with a third independent reviewer. Reasons for exclusion were recorded. Included studies proceeded to the data extraction phase following full-text screening.

#### Data collection process and data items

Data items that were extracted from the records included study, author, year, study design, participants included, study duration, participant demographics, follow-up (if any) and outcome measures.

### Risk of bias in individual studies

Risk of bias was assessed using the Cochrane risk of bias tool for RCTs, following the Cochrane handbook. [14] For non-RCTs, the Downs and Black [15] tool was used to assess the risk of bias. Additional details on the risk of bias assessments are provided in the Supplementary Methods.

### Data synthesis

This systematic review did not include a meta-analysis; effect was measured by a narrative synthesis. Although both RCTs and non RCTs were eligible for inclusion, RCTs are discussed in more detail owing to a more robust study design for reporting purposes.

## Results

### Study selection and exclusion

**Fig. 1:**
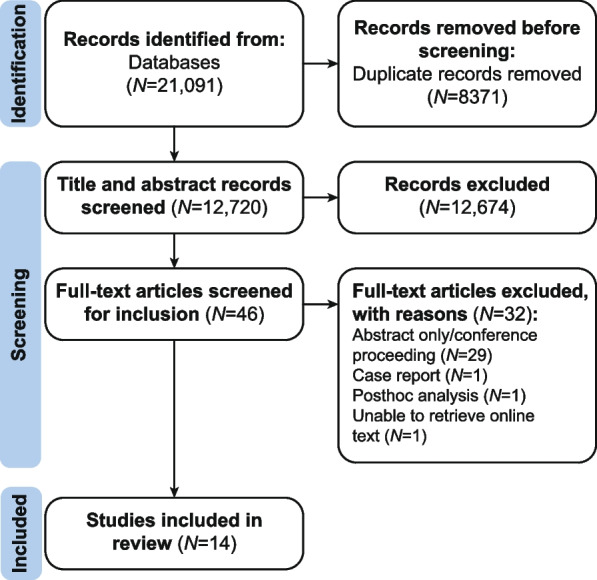
PRISMA flow diagram of the studies included in this review**.** Databases that were searched included PubMed/Medline, Embase, Biosis/Ovid, Web of Science, and Cochrane Library. N, number of studies

The final search yielded 21,091 articles from five databases (Fig. 1). After removing duplicates (*N* = 8371), 12,720 titles and abstracts were screened, 12,674 records were excluded, and 46 articles were included for full-text screening. Of these, 32 articles were excluded for the following reasons: abstract only/conference proceeding (*N* = 29), case report (*N* = 1), post hoc analysis (*N* = 1), and unable to retrieve online text (*N* = 1). Fourteen studies were included in the review. [16–29].

### Study characteristics

Intervention details of the included RCTs and non-RCTs are shown in Table 1 and Supplementary Table S2, respectively. Of the 14 studies, 10 were RCTs [16–25], 3 were retrospective studies [26–28] and 1 was a post hoc analysis of an RCT originally designed for atherosclerosis. [29] IL-1β inhibitors included in the RCTs were canakinumab (*N* = 3) [19–21], anakinra (*N* = 2) [16, 18] and rilonacept (*N* = 5). [17, 22–25] IL-1β inhibitors in non-RCTs included canakinumab (*N* = 1) [29] and anakinra (*N* = 3). [26–28] The intervention duration ranged from 3 days [25] to 16 weeks. [17, 21–24] Overall, the RCTs (*N* = 10) included 3446 patients, retrospective studies (*N* = 3) included 166 patients (159 patients had a previous history of gout), and the post hoc analysis (*N* = 1) included 10,059 patients (762 patients had a previous history of gout). The most common primary endpoints in the RCTs were the presence/number of gout flares (*N* = 5) and pain (*N* = 5).

**Table 1:** Intervention details of the RCTs

Author	Study design (duration)	Number of patients by treatment group	Intervention and comparator	Primary endpoint
**CAN (*N* = 3)**				
Schlesinger et al., 2011a [20]	Adaptive single-dose, single-blind, active-controlled study (8 wk)	CAN: *n* = 143 TA:* n* = 57	**Intervention:** One dose of CAN (10, 25, 50, 90, or 150 mg) and saline on day 1 **Comparator:** One dose of TA 40 mg and PBO on day 1	Pain (note: pain was the primary endpoint of the original study but is also reported in this study)
Schlesinger et al. 2011b [21]	Dose-ranging, multicentre, double-blind, double-dummy, active-controlled study (16 wk + 8-wk FU)	CAN 25‒300 mg: *n* = 270 CAN q4wk: *n* = 53 CLC: *n* = 108	**Intervention:** One dose of CAN (25, 50, 100, 200 mg, 300 mg) or CAN q4wk **Comparator:** CLC 0.5 mg q.d	CAN dose producing equivalent efficacy to CLC 0.5 mg (mean number of GFs PP)
Schlesinger et al., 2012 [19]	2 multicentre, active-controlled, double-blind, parallel-group, double-dummy, phase 3 studies (12 wk + 12-wk ext)	CAN: *n* = 225 TA:* n* = 229	**Intervention:** CAN 150 mg; PBO matching for each GF **Comparator:** TA 40 mg; PBO matching for each GF	Pain intensity in the most affected joint at 72 h post-dose and time to first new GF
**ANK (*N* = 2)**				
Janssen et al., 2019 [16]	Randomised, double-blind, double-dummy, active comparator, PBO-controlled trial (5 d + 2-d safety FU)	TaU: *n* = 45 ANK*: n* = 43	**Intervention:** 5-d treatment with ANK (100 mg q.d.) + PBO up to t.i.d. (CLC), b.i.d. (NAP) or q.d. (PRED) **Comparator:** TaU (0.5 mg up to t.i.d. for CLC; 500 mg up to b.i.d. for NAP; 35 mg q.d. for 5 d for PRED) + PBO q.d. for 5 d	Mean change in pain in the most affected joint from BL to the average of pain scores at days 2‒4 with a prespecified non-inferiority margin of 0.4
Saag et al., 2021 [18]	Randomised, double-blind, double-dummy, active-control, multicentre trial (15 d + 52-wk post-randomisation ext)	TA: *n* = 55 ANK:* n* = 110	**Intervention:** ANK 100 mg q.d. for 5 d/ANK 200 mg q.d. for 5 d **Comparator:** TA 40 mg single injection on day 1	Change in pain intensity from BL to 24–72 h
**RL (*N* = 5)**				
Mitha et al., 2013 [17]	Randomised, double-blind, PBO-controlled, phase 3 study (16 wk + 5-wk safety FU)	PBO: *n* = 82 RL: *n* = 166	**Intervention:** RL 80 mg/160 mg. q.w with loading doses of RL 160 mg (80-mg group) & 320 mg (160-mg group) on day 1, followed by 15 q.w. doses alongside AP 300 mg q.d **Comparator:** PBO q.w. Loading doses on treatment day 1 followed by 15 q.w. Patients also initiated on AP 300 mg q.d	Mean number of GFs PP up to week 16
Schumacher et al., 2012a [23]	Phase 2, randomised, double-blind, PBO-controlled trial (16-wk + 6-wk FU)	PBO: *n* = 42 RL: *n* = 41	**Intervention:** RL 160 mg q.w. (loading dose: RL 320 mg) and AP 300 mg q.d **Comparator:** PBO q.w. and AP 300 mg q.d	Number of GFs PP through week 12
Schumacher et al., 2012b [22]	Phase 3, randomised, double-blind, PBO-controlled, confirmatory efficacy study (16-wk + 4-wk safety FU)	PBO: *n* = 79 RL: *n* = 161	**Intervention:** RL 80 mg/160 mg q.w. Loading doses of RL 160 mg (80-mg group), 320 mg (160-mg group) on treatment day 1, alongside AP q.d **Comparator:** PBO q.w. Loading doses of PBO were administered on treatment day 1, alongside AP q.d	Mean number of GFs PP through week 16
Sundy et al., 2014 [24]	Phase 3, randomised, double-blind, PBO-controlled trial (16-wk + 4-wk safety FU)	PBO: *n* = 330 RL: *n* = 985	**Intervention:** RL 160 mg q.w. Loading dose of RL 320 mg was administered in 2 equal volumes on day 1 **Comparator:** Loading dose of PBO was administered in 2 equal volumes on day 1, followed by 15 q.w. doses of PBO	Safety (AE, SAE, and clinical laboratory variables) over 20 weeks
Terkeltaub et al., 2013 [25]	Phase 3, randomised, double blind, double-dummy, active- and PBO-controlled study (3‒9 d + safety FU on d 31)	PBO + IND: *n* = 75 RL + IND: *n* = 74 RL + PBO: *n* = 73	**Intervention:** RL 320 mg at BL + IND (50 mg t.i.d. for 3 d [then 25 mg t.i.d. for up to 9 d]) or RL 320 mg at BL + PBO t.i.d. for 3 d (then PBO t.i.d. for up to 9 d) **Comparator:** PBO at BL + IND 50 mg t.i.d. for 3 d (then 25 mg t.i.d. for up to 9 d)	Pain in the index joint at 24, 48 and 72 h

*AE* adverse event, *ANK* anakinra, *AP* allopurinol, *b.i.d* twice daily, *BL* baseline, *CAN* canakinumab, *CLC* colchicine, *d* days(s), *ext* extension, *FU* follow-up, *GF* gout flare, *h* hour(s), *IND* indomethacin, *n* number of patients in group, *N* number of studies, *NAP* naproxen, *PBO* placebo, *PP* per patient, *PRED* prednisone, *q.d* once daily, *q.w* once weekly, *q4wk* every 4 weeks, *RCT* randomised controlled trial, *RL* rilonacept, *SAE* serious adverse event, *t.i.d* three times daily, *TA* triamcinolone acetonide, *TaU* treatment as usual, *wk* week(s)

### Patient and disease characteristics in the included studies

Baseline demographic and disease characteristics are detailed in Table 2 and Supplementary Table S3. Most patients included in the RCTs were male (individual group range: 82.8%‒100%); the mean age ranged from 48.6 to 63.4 years (individual group range). Where reported in the RCTs, patients within individual study groups had an average of 3.6‒7.1 gout flares per year/in the previous year, 4.9%‒38.9% had tophi and average disease duration, where reported, was 7.7‒12.6 years (Table 2).

**Table 2:** Baseline demographic and disease characteristics of the RCTs

Author	Sex, male (%)	Age range, years, mean (SD)	Disease duration, years, mean (SD)	Number of GFs, mean (SD)	Presence of tophi, %	Reason for prescribing IL-1β inhibitors
**CAN (*N* = 4)**						
Schlesinger et al., 2011a [20]	CAN: 82.8%‒100.0% TA: 96.5%	CAN: 49.9 (11.1) to 54.9 (10.8) TA: 52.4 (11.6)	NR	**Previous year:** CAN: 3.9 (2.6) to 6.8 (8.1) TA: 6.5 (9.9)	NR	NR
Schlesinger et al., 2011b [21]	CAN 25‒300 mg: 88.9%‒100.0% CAN q4wk: 92.5% CLC: 93.5%	CAN 25‒300 mg: 50.7 (9.7) to 54.4 (12.2) CAN q4wk: 52.8 (10.4) CLC: 52.4 (10.7)	** > 10-years, N (%):** CAN 25‒300 mg: 15 (27.8) to 24 (43.6) CAN q4wk: 18 (34.0) CLC: 33 (30.6)	**Previous year:** CAN 25‒300 mg: 3.6 (2.3) to 4.7 (4.5) CAN q4wk: 4.4 (4.1) CLC: 4.3 (3.8)	NR	NR
Schlesinger et al., 2012 [19]	CAN: 89.3% TA: 93.0%	CAN: 52.3 (11.8) TA: 53.6 (11.5)	** > 10-years, N (%):** CAN: 69 (30.7) TA: 96 (41.9)	**Previous year:** CAN: 6.5 (5.6) TA: 6.5 (4.8)	CAN: 28.4% TA: 29.7%	Having contraindications for, intolerance of, or unresponsiveness to NSAIDs and/or CLC
**ANK (*N* = 2)**						
Janssen et al., 2019 [16]	TaU: 93.3% ANK: 95.3%	TaU: 59.9 (12.7) ANK: 63.4 (12.9)	NR	NR	NR	NR
Saag et al., 2021 [18]	TA: 87.3% ANK: 85.7%‒87.0%	**Median (range):** TA: 56.0 (30‒83) ANK: 53.5 (25‒79) to 54.0 (27‒78)	TA: 7.7 (7.6) ANK: 8.6 (7.7) to 9.7 (8.8)	**Previous year:** TA: 4.4 (2.0) ANK: 4.4 (1.7) to 4.6 (3.4)	TA: 38.2% ANK: 30.4%‒38.9%	Patients had to have non-responsiveness to NSAIDs and CLC or were contraindicated to them
**RL (*N* = 5)**						
Mitha et al., 2013 [17]	PBO: 93.9% RL: 91.7%‒93.9%	PBO: 51.7 (12.9) RL: 49.0 (11.8) to 52.6 (11.5)	PBO: 9.6 (8.8) RL: 8.7 (7.0) to 12.6 (10.3)	**P/Y:** PBO: 7.1 (6.9) RL: 6.8 (5.4) to 7.0 (5.7)	PBO: 22.0% RL: 25.0%‒25.6%	NR
Schumacher et al., 2012a [23]	PBO: 95.2% RL: 97.6%	PBO: 50.1 (11.6) RL: 51.9 (10.6)	PBO: 8.6 (7.0) RL: 10.7 (9.1)	**Previous year:** PBO: 4.4 (4.0) RL: 4.7 (3.2)	PBO: 14.3% RL: 4.9%	NR
Schumacher et al., 2012b [22]	PBO: 96.2% RL: 88.8%‒93.8%	PBO: 52.2 (13.6) RL: 51.9 (11.6) to 52.9 (12.5)	PBO: 11.2 (9.4) RL: 9.1 (8.3) to 10.0 (8.3)	**P/Y:** PBO: 4.6 (3.6) RL: 4.5 (3.6) to 4.6 (2.9)	PBO: 10.1% RL: 9.9%‒12.5%	NR
Sundy et al., 2014 [24]	PBO: 90.0% RL: 87.0%	PBO: 52.4 (10.6) RL: 52.8 (11.5)	PBO: 10.6 (8.4) RL: 10.7 (9.6)	**P/Y:** PBO: 6.1 (7.2) RL: 6.0 (6.3)	PBO: 30.9% RL: 28.3%	NR
Terkeltaub et al., 2013 [25]	PBO + IND: 94.7% RL + IND: 95.9% RL + PBO: 91.8%	PBO + IND: 51.3 (10.9) RL + IND: 48.6 (10.0) RL + PBO: 51.0 (10.8)	PBO + IND: 8.8 (6.7) RL + IND: 11.0 (7.9) RL + PBO: 10.2 (9.9)	**P/Y:** PBO + IND: 4.8 (5.2) RL + IND: 5.5 (5.3) RL + PBO: 5.2 (4.8)	PBO + IND: 13.3% RL + IND: 16.2% RL + PBO: 17.8%	NR

*ANK* anakinra, *CAN* canakinumab, *CLC* colchicine, *GF* gout flare, *h* hour(s), *IND* indomethacin, *N* number of studies, *NR* not reported, *PBO* placebo, *P/Y* per year, *q4wk* every four weeks, *RCT* randomised controlled trial, *RL* rilonacept, *SD* standard deviation, *TA* triamcinolone acetonide, *TaU* treatment as usual

### Narrative synthesis

#### Effects of IL-1β inhibitors on pain and gout flares

The results of the efficacy outcomes for RCTs and non-RCTs are detailed in Table 3 and Supplementary Table S4, respectively.

**Table 3:** Efficacy results of the RCTs

Author	Number of GFs	Severity of GFs or pain	Duration of GFs and/or time between GFs	Synovitis	Other outcomes
**CAN (*N* = 3)**					
Schlesinger et al., 2011a [20]	NR	**Primary endpoint:** % of patients with no/mild pain was numerically greater in most CAN groups vs TA from 24 to 72 h; the difference was significant for the 150-mg group The reduction in pain intensity from BL was also greater for CAN 150 mg vs TA from 48 h to 7 d	NR	**Primary endpoint:** All treatments reduced visible inflammation in the target joint by 72-h. At 72-h, CAN 150 mg had a lower score for tenderness and swelling vs TA; the difference remained significant at 7 d. Erythema was absent in 74.1% (CAN 150 mg) and 69.6% (TA) at 72-h and in 96.3% (CAN 150 mg) & 83.9% (TA) at 7 d	NR
Schlesinger et al., 2011b [21]	**Primary endpoint:** **Mean GFs PP (least-squares mean [SE]; ANCOVA):** CAN 25 mg: 0.5 (0.2) CAN 50 mg: 0.5 (0.2) CAN 100 mg: 0.2 (0.2) CAN 200 mg: 0.4 (0.2) CAN 300 mg: 0.2 (0.2) CAN q4wk: 0.7 (0.2) CLC 0.5 mg: 0.8 (0.1) Significant for CAN 100 mg and 300 mg vs CLC 0.5 mg	NR	**Time to first GF (HR [95% CI]):** CAN 25 mg: 0.6 (0.3‒1.0) CAN 50 mg: 0.3 (0.2‒0.7) CAN 100 mg: 0.3 (0.1‒0.6) CAN 200 mg: 0.4 (0.2‒0.7) CAN 300 mg: 0.3 (0.1‒0.6) CAN q4wk: 0.3 (0.2‒0.7) **Average duration of GFs (least-squares mean [SD]):** CAN 25 mg: 4.6 (1.0) CAN 50 mg: 3.7 (1.2) CAN 100 mg: 2.8 (1.4) CAN 200 mg: 3.6 (1.3) CAN 300 mg: 3.1 (1.3) CAN q4wk: 3.3 (1.2) CLC: 5.1 (0.6)	NR	**Patients experiencing ≥ 1 GF (n [%]):** CAN 25 mg: 15 (27.3) CAN 50 mg: 9 (16.7) CAN 100 mg: 8 (14.8) CAN 200 mg: 10 (18.5) CAN 300 mg: 8 (15.1) CAN q4wk: 9 (16.7) CLC: 48 (44.4) All CAN patients were significantly less likely to experience ≥ 1 GF vs CLC patients (OR: 0.22–0.47)
Schlesinger et al., 2012 [19]	** ≥ 1 GF:** CAN: 16.0% TA: 35.8% (OR: 0.34) **Mean number of new GFs:** CAN: 0.19 TA: 0.51 (rate ratio: 0.37) ** ≥ 2 GFs:** CAN: 2.7% TA: 11.4% **Median time to new GFs:** CAN: > 168 d TA: 131 d	**Primary endpoint:** **Mean pain scores at 72 h:** CAN: 25.0 mm TA: 35.7 mm MD: ‒10.7 mm (95% CI: ‒15.4 to ‒6.0)	CAN delayed time to first new GF and reduced new GF risk over the 12-wk period by 62% vs TA	**72 h (OR vs TA [95% CI]):** **Tenderness:** CAN: 2.2 (1.5‒3.1) **Swelling:** CAN: 1.7 (1.2‒2.5) **Erythema:** CAN: 0.6 (0.4‒0.9)	**Taking rescue medication:** CAN: 37.3% (oral steroids: 11.1%) TA: 54.6% (oral steroids: 23.6%)
**ANK (*N* = 2)**					
Janssen et al., 2019 [16]	NR	**Primary endpoint:** Pain scores decreased to a similar extent in both groups. Estimated marginal mean difference between treatment arms: ‒0.13 points in favour of ANK. The upper 95% CI (-0.44 to 0.18) did not surpass the NI margin of 0.4	NR	For all the secondary outcomes, pattern of change was similar for ANK and TaU over 5 d	During the first 7 d, numerically more patients in ANK (*n* = 20; 46.5%) vs TaU (*n* = 16; 35.6%), took pain-relieving medication. After 2 d, numerically more patients on ANK achieved ≥ 50% decrease in NRS pain scores (OR: 1.4; 95% CI: 0.5‒3.7) vs TaU. On day 3‒5, ORs were in favour for ANK (only significant on day 3)
Saag et al., 2021 [18]	**Treated for 1 GF:** TA: *n* = 54 ANK 100 mg: *n* = 55 ANK 200 mg: *n* = 52 **Treated for 2 GFs:** TA: *n* = 17 ANK 100 mg: *n* = 23 ANK 200 mg: *n* = 21 **Treated for 3 GFs:** TA: *n* = 5 ANK 100 mg and 200 mg: *n* = 13	**Primary endpoint:** **Change in pain from BL to 24‒72 h after first GF (mean [95% CI]):** TA: − 39.4 (− 46.8 to − 32.0) ANK 100 mg: − 41.8 (− 48.9 to − 34.8) ANK 200 mg: − 40.7 (− 47.9 to − 33.4) Pain intensity for the first GF at 6, 12, 18, 24, 36, 48, and 72 h and 5, 6, 7, and 8 d was similar in ANK and TA groups Across treatment arms, reduction in pain intensity for the second and third GF was similar to the first GF	**Median time to pain resolution for the first GF:** ANK total: 120.5 h TA: 167.5 h (HR: 1.3 [95% CI: 0.9‒1.9]) **Median time to response:** ANK total: 46.7 h TA 40 mg: 47.6 h (HR: 1.2 [95% CI: 0.8‒1.7]) **Median time to onset of effect for the first GF:** ANK total: 17.8 h TA: 22.3 h (HR: 1.1 [95% CI: − 0.8 to 1.6]) **Resolution of pain by day 15:** ANK total: 70 (63.6%) TA 40 mg: 36 (65.5%)	**Mean physician assessment of tenderness and swelling at 72 h:** ANK total: − 0.5 (95% CI: − 0.7 to − 0.2; significant) TA: − 0.3 (95% CI: − 0.6 to − 0.1) and was also better for swelling on day 8 (− 0.3 [95% CI: − 0.6 to − 0.1]) Significantly less presence of erythema was reported in ANK total vs TA group at 72 h (OR: 0.5 [95% CI: 0.2‒1.0])	Between BL and day 15, 44.5% of patients in the ANK total group and 47.3% in the TA group received rescue medication
**RL (*N* = 5)**					
Mitha et al., 2013 [17]	**Primary endpoint:** **Number of GFs at week 16:** PBO: 101 RL 80 mg: 29 RL 160 mg: 28 RL 160 mg had significantly fewer GFs PP (0.3; 95% CI: 0.2‒0.5) vs PBO (1.2; 95% CI: 0.9‒1.6), a 72.6% rate reduction (95% CI: 58.4%‒82.0%) **Wk-16 GF days PP:** PBO: mean of 11.2 (95% CI: 6.6‒15.8) RL 80 mg: 4.3 (95% CI: 0.5‒8.1) RL 160 mg: 1.9 (95% CI: 0.6‒3.1)	Treatment with RL resulted in significantly fewer days PP with a pain severity score ≥ 5 vs PBO. For the 80-mg group, this reduction was from 4.3 d (95% CI: 2.6‒6.0) with PBO to 1.7 d (95% CI: 0.0‒3.5), and for RL 160 mg, the reduction was to 0.9 d (95% CI: 0.3‒1.5)	The estimated median time to first GF in the PBO group was 34 d, significantly earlier than for either of the RL groups. Median time could not be estimated as < 50% of RL patients reported a GF	NR	**The RR of ≥ 1 GF over 16 wk:** RL 80 mg: 0.5 (95% CI: 0.3‒0.7); risk reduction: 54.3% (95% CI: 30.8%‒69.9%) RL 160 mg: 0.4 (95% CI: 0.2‒0.6); risk reduction: 63.5% (95% CI: 41.9‒77.1) **Patients with ≥ 1 GF:** PBO: 56.1% RL 80 mg: 25.6% RL 160 mg: 20.5% **Patients with ≥ 2 GFs:** PBO: 32.9% RL 80 mg: 8.5% RL 160 mg: 6.0%
Schumacher et al., 2012a [23]	**Primary endpoint:** **Mean number of GFs PP:** PBO: 0.8 (33 GFs) RL: 0.2 (6 GFs) 81% decrease in GFs with RL with decrease maintained at ext and FU Fewer GFs in RL groups as early as 4 wk	**Days with pain score ≥ 5 PP (mean [SD]):** PBO: 2.0 (4.5) RL: 0.2 (0.8)	**GF at week 4:** PBO: 26.2% RL: 4.9% **Median time to first GF:** PBO: 77 d RL: N/A **GF days PP (mean [SD]):** PBO: 5.2 (8.0) RL: 1.4 (5.2)	NR	**Patients with ≥ 1 GF (n [%]):** PBO: 19 (45.2) RL: 6 (14.6)
Schumacher et al., 2012b [22]	**Primary endpoint:** **Number of GFs over 16-wk:** PBO: 84 RL 80 mg: 23 RL 160 mg: 17 **Mean number of GFs PP:** PBO: 1.1 (95% CI: 0.7–1.4) RL 80 mg: 0.3 (95% CI: 0.1–0.5) RL 160 mg: 0.2 (95% CI: 0.1–0.3) **Reduction in GFs vs PBO:** RL 80 mg: 73.0% (95% CI: 57.1–83.0%) RL 160 mg: 80.0% (95% CI: 66.3–88.1%) Observed as early as 4 wk	**Days PP with pain severity score ≥ 5 (mean [95% CI]):** PBO: 2.1 (1.4‒2.8) RL 80 mg: 0.9 (0.0‒1.7) RL 160 mg: 0.4 (0.1‒0.6)	**GF days PP (mean [95% CI]):** PBO: 5.5 (3.3‒7.7) RL 80 mg: 2.4 (0.0‒4.9) RL 160 mg: 1.0 (0.3‒1.6)	NR	**Patients using rescue medication up to week 16:** PBO: 54.4% RL 80 mg: 25.0% RL 160 mg: 23.5% **RR for ≥ 1 GFs:** RL 80 mg: 0.4 (95% CI 0.2–0.7) RL 160 mg: 0.4 (95% CI 0.2–0.6) **Percentage of patients > 1 GFs:** PBO: 31.6% RL 80 mg: 5.0% RL 160 mg: 3.8%
Sundy et al., 2014 [24]	**Mean number of GFs PP at week 16:** RL: 70.3% reduction vs PBO: 1.7 (95% CI: 1.4‒2.0) to 0.5 [95% CI: 0.4‒0.6] ** ≥ 1 GF by week 16:** PBO: 51.1% RL: 25.7% (49.6% reduction) ** ≥ 2 GFs by week 16:** PBO: 34.7% RL: 11.7% (66.4% reduction)	NR	**Median time to first GF:** PBO: 87 d RL: N/A as < 50% of patients (25.7%) reported GFs **Total number of GF days PP at week 16:** PBO: 7.7 (95% CI: 6.4‒9.0) RL: 2.7 (95% CI: 2.2‒3.2, 64.9% reduction)	NR	**At week 16, GFs PP with tophi (mean [SD]):** PBO: 2.1 (2.9) RL: 0.9 (1.6) **Without tophi:** PBO: 1.6 (2.6) RL: 0.4 (0.9)
Terkeltaub et al., 2013 [25]	NR	**Primary endpoint:** All groups had significant pain reductions from BL when averaged at 24-, 48- and 72 h RL + IND mean pain reduction (1.6 points) was not significantly greater than IND alone (1.4 points): least squares mean difference: ‒0.1 (95% CI: ‒0.4 to 0.2) The difference between IND and RL favoured IND	NR	NR	**Patients taking rescue medication at > 24‒48 h:** RL + IND: 3.0% IND: 4.3% Mean change in pain at 24, 48, and 72 h were similar between groups except IND alone was significantly superior to RL alone at all time points

*ANCOVA* analysis of covariance, *ANK* anakinra, *BL* baseline, *CAN* canakinumab, *CI* confidence interval, *CLC* colchicine, *d* day(s), *FU* follow-up, *GF* gout flare, *h* hour(s), *HR* hazard ratio, *IND* indomethacin, *MD* mean difference, *n* number of patients in group, *N* number of studies, *NI* non-inferior, *NR* not reported, *NRS* numerical rating scale, *OR* odds ratio, *q4wk* every 4 weeks, *PBO* placebo, *PP* per patient, *RCT* randomised controlled trial, *RL* rilonacept, *RR* risk ratio, *SD* standard deviation, *SE* standard error, *TA* triamcinolone acetonide, *TaU* treatment as usual, *wk* week(s)

##### Canakinumab RCTs (N = 3) [19–21]

Two RCTs reported using canakinumab to treat gout flares [19, 20] and one reported using canakinumab to reduce gout flare frequency. [21] Comparator medications included triamcinolone acetonide (TA) [19, 20] and colchicine. [21] Where assessed, patients receiving canakinumab had fewer gout flares during the intervention than those receiving the comparators, with more patients reporting less severe pain with canakinumab (compared to comparators). The canakinumab arms had reduced signs of synovitis, where assessed [19, 20], and took less rescue medication during the intervention compared to the comparator arms.

##### Anakinra RCTs (N = 2) [16, 18]

Two RCTs reported using anakinra to treat gout flares. [16, 18] Comparator medications included TA [18] or treatment as usual (colchicine, naproxen, or prednisone). [16] Where assessed, the proportion of patients treated for one gout flare was similar between the anakinra and comparator arms, with more patients in the anakinra arm treated for multiple gout flares. There were no differences in change in pain between the anakinra and comparator arms, suggesting that anakinra is not inferior to the comparator. In one study, synovitis was not different between the arms [16], whereas in the other study, anakinra was better than the comparator for physician’s assessment of tenderness and swelling, and less erythema was reported in the anakinra arm. [18] There were no differences between the anakinra and comparator arms for use of rescue medication.

##### Rilonacept RCTs (N = 5) [17, 22–25]

One RCT reported using rilonacept to treat gout flares [25], whereas four reported using rilonacept to reduce gout flare frequency. [17, 22–24] All comparator medications were reported as placebo [17, 22–24], except for one study, which used indomethacin and a placebo. [25] Where assessed, the average number and proportion of gout flares, and the proportion of patients experiencing gout flares, were lower with rilonacept than with the comparator, and patients treated with rilonacept experienced gout flares later in the treatment course. Similarly, where assessed, patients in the rilonacept arms had less pain compared with the comparator arms, except for one study where the comparator was favoured. [25] Synovitis was not assessed in any rilonacept RCT. Where assessed, the rilonacept arms reported less rescue medication use compared with the comparator arms, with the exception of one study, which reported no differences. [25].

##### Non-RCTs [26–29]

In the retrospective studies, anakinra resulted in significant/good pain improvement in 67.0%‒90.0% of patients. [26, 28] In the post hoc analysis, canakinumab reduced the risk of a gout flare during the follow-up period of the trial by 52%. [29].

#### Effects of IL-1β inhibitors on safety and additional outcomes

The results of the safety and additional outcomes are detailed in Supplementary Tables S5 and S6, respectively.

##### Canakinumab RCTs (N = 3) [19–21]

**Safety** - Overall, adverse events (AEs) occurred in 41.3%‒66.2% (canakinumab) and 42.1%‒53.7% (comparator) of patients in the canakinumab RCTs. The incidence of AEs was similar between arms, except for one study where the AE incidence in the canakinumab arm was 66.2% versus 52.8% in the comparator arm. [19] AEs were generally mild or moderate in severity with no evidence of a dose–response relationship. Common AEs were hypertension (9.3%‒10.9%), arthralgia (7.4%‒9.3%) and headache (5.7%‒11.3%) in the canakinumab arms and hypertension (5.7%) and headache (5.6%) in the comparator arms. In all studies, the incidence of infections in the canakinumab arms ranged from 7.0%‒20.4% versus 7.0%‒12.2% in the comparator arms. [19–21] Where reported, serious adverse events (SAEs) occurred in 0.0%‒7.6% of canakinumab-treated patients and 0.0%‒5.6% of comparator-treated patients. AEs leading to discontinuation were reported in 0.0%‒1.2% (canakinumab) and 0.0%‒1.9% (comparator) of patients. Three deaths were reported in the three RCTs (*N* = 1, canakinumab arm; *N* = 2, comparator arms).

**Additional outcomes** - Where assessed, canakinumab had positive benefits on QoL and reduced C-reactive protein (CRP) concentration to a greater extent compared to the comparator. Furthermore, canakinumab generally resulted in a greater global response to treatment than the comparator.

##### Anakinra RCTs (N = 2) [16, 18]

**Safety** - Overall, AEs occurred in 34.9%‒55.8% (anakinra) and 40.7%‒46.7% (comparator) of patients, where reported. In both RCTs, AE incidence was similar in both arms. In one study, the most common AEs were “other AEs” (24.3%) and musculoskeletal pain (16.2%) in the anakinra arm and “other AEs” (20.4%) and diarrhoea (18.4%) in the comparator arm. [16] In the other study, hypertriglyceridemia, neutropenia, and various injection site reactions were the most common AEs in the anakinra arm, whereas headache was the most common AE in the comparator arms. [18] In one study, the incidence of infection was 2.7% in the anakinra arm and 2.0% in the comparator arm. [16] AEs were mostly mild or moderate in severity. In one study [18], SAEs occurred in 1.9%‒7.3% (anakinra) and 0.0% (comparator) of patients. Overall, 1.8%‒3.8% (anakinra) and 5.6% (comparator) of patients had AEs which led to discontinuation. No deaths were reported in the two studies.

**Additional outcomes** - No studies using anakinra reported the effects on QoL. The effects of anakinra on CRP were mixed between the two studies, with one reporting no differences versus the comparator arm [16] and the other reporting reduced CRP levels with anakinra versus the comparator. [18] Both studies reported on global assessment of treatment response, with treatment response being greater in the anakinra arm in one study [18] and no differences reported between arms in the other study. [16].

##### Rilonacept RCTs (N = 5) [17, 22–25]

**Safety** - AEs occurred in 36.0%‒68.3% (rilonacept) and 29.9%‒61.0% (comparator) of patients. In all studies, the incidence of AEs was similar between rilonacept and comparator arms. Common AEs were injection/infusion site reactions (8.8%‒19.8%), upper respiratory tract infection (9.8%‒12.2%) and headache (5.5%‒9.3%) in the rilonacept arms and upper respiratory tract infection (9.5%‒12.2%), joint-related signs and symptoms (9.5%) and headache (7.8%) in the comparator arms. In four studies, the incidence of infections and infestations ranged from 14.6%‒28.0% in the rilonacept arm and 19.1%‒26.2% in the comparator arm. [17, 22–24] SAEs occurred in 0.0%‒6.1% (rilonacept) and 0.0‒4.9% (comparator) of patients, where reported. SAE incidence was similar between the rilonacept and comparator arms. AEs leading to discontinuation were reported in 1.3%‒5.0% (rilonacept) and 0.0%‒7.1% (comparator) of patients. Seven deaths were reported across the studies (*N* = 4, rilonacept arm; *N* = 3, comparator arm).

**Additional outcomes** - No rilonacept studies reported on the effects on QoL. One study reported the effects of rilonacept on high-sensitivity CRP and reported that rilonacept reduced CRP to a greater extent than the comparator. [25].

##### Non-RCTs (N = 4) [26–29]

**Safety** - Few AEs were reported, and anakinra was well tolerated in the three retrospective anakinra studies. AEs reported included leukopenia and infectious complications. SAEs were not detailed in the retrospective studies. The post hoc analysis on canakinumab did not report on AEs. [29].

**Additional outcomes** - In these studies, anakinra reduced CRP. In the post hoc analysis, canakinumab reduced CRP.

### Risk of bias

**Fig. 2:**
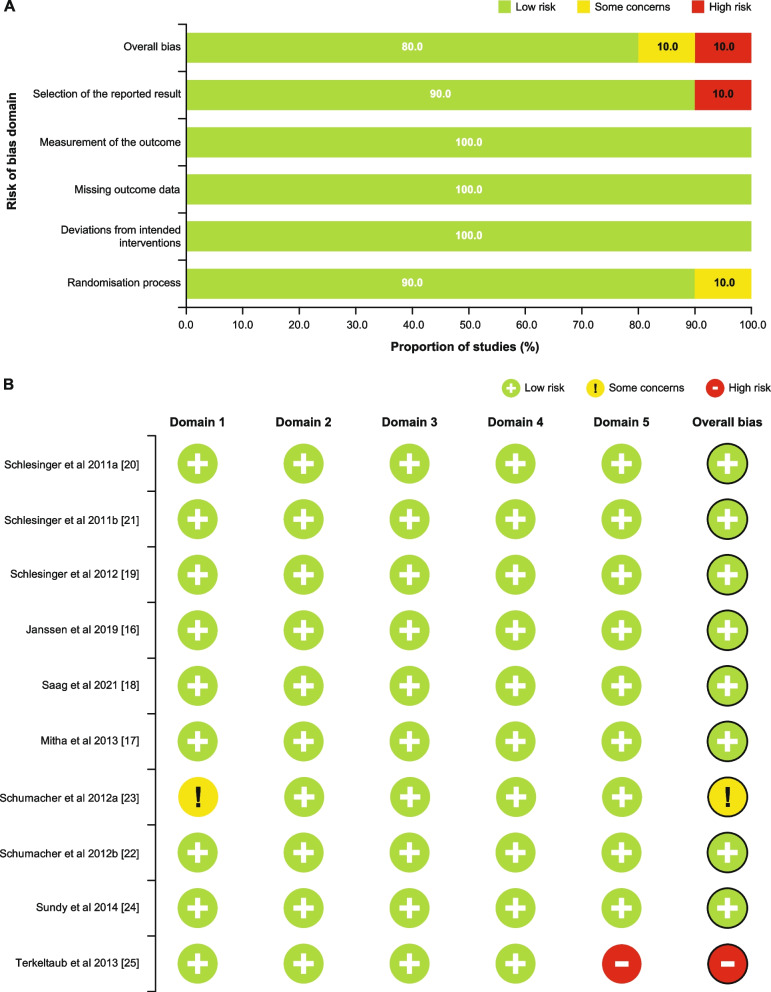
Summary of the Cochrane risk of bias assessment in included RCTs (**A**) overall and (**B**) in individual studies**.** Domain 1, randomisation process; domain 2, deviations from the intended interventions; domain 3, missing outcome data; domain 4, measurement of the outcome; domain 5, selection of the reported result. RCT, randomised controlled trial

Overall, 80.0% of the articles were at low risk of bias, 10.0% had some concerns for risk of bias, and 10% had a high risk of bias (Fig. 2A). The main source for high risk of bias was ‘selection of the reported result’ (10.0%) [25] and for some concerns for risk of bias was ‘randomisation process’ (10.0%). [23] The Cochrane risk of bias results for individual studies are shown in Fig. 2B.

The results of the Downs and Black risk of bias are shown in Supplementary Table S7. The three retrospective studies had a high degree of bias, with total scores ranging from 5‒14 (higher scores indicate a lower risk of bias). All assessed domains contributed to the high risk of bias scores. The post-hoc analysis had a relatively lower risk of bias, with a total score of 24.

## Discussion

This systematic review assessed the effectiveness of IL-1β inhibitors for the management of gout flares by examining the accumulated evidence of studies published between 2011 and 2022 and represents the first systematic review of this topic in nearly a decade.

Our results underline the potential benefit of IL-1β inhibitors for treating gout flares in patients who fail or cannot tolerate standard therapy. A total of 14 studies (10 RCTs, 3 retrospective studies and 1 post hoc analysis) were considered and included canakinumab, anakinra, and rilonacept therapies. No studies using gevokizumab, another IL-1β inhibitor which may have potential to treat gout [30], were retrieved from our search, which is consistent with a 2018 report. [31] Current guidelines conditionally recommend the use of IL-1β inhibitors only when other therapies (colchicine, NSAIDs and glucocorticoids) are ineffective, poorly tolerated, or contraindicated. [5, 7, 9, 10] .

Mixed evidence is available for the three included therapies for their effectiveness in improving gout-related pain associated with flares. Overall, canakinumab and rilonacept were more effective in reducing gout-related pain compared to their respective comparators. However, one rilonacept study reported no positive benefits of rilonacept over the comparator for reducing pain. [25] For the anakinra studies, although most were not designed to assess non-inferiority, the response to treatment between anakinra and active comparators was broadly comparable for reducing gout-related pain. Controlling gout-associated pain, a debilitating symptom of gout flares, is the most important therapeutic goal when treating gout. [2, 4] Furthermore, the Outcome Measures in Rheumatology (OMERACT) group cites pain as an important outcome in acute gout flares [32], and patients have reported severe pain as the most important symptom of gout. [33].

This systematic review also provides insights into the effectiveness of IL-1β inhibitors on the occurrence and frequency of gout flares. Both canakinumab and rilonacept generally resulted in improvements in gout flare-related outcomes and associated signs of synovitis compared to their respective comparators, whereas anakinra showed mixed effects compared with the respective comparator, indicating that more RCTs involving anakinra are warranted for better understanding. The discordance between anakinra’s effects compared to canakinumab and rilonacept may be due to the differing mechanisms of action between the treatments; canakinumab selectively inhibit IL-1β, while anakinra inhibits IL-1 receptor type 1. Although rilonacept also inhibits IL-1α and IL-1 receptor antagonist protein, it does so with less affinity compared to its ability to bind and neutralize IL-1β. Differences in study design, especially with regards to the choice of comparator drug, selection of the primary outcome measure, and the time at which the primary endpoint is assessed may also contribute to the discordance observed between the effects of each treatment. Additionally, the half-life of anakinra is shorter than the other agents (hours versus weeks) which may have implications for both the effects of the agent and the flexibility practitioners have with its use. Treating gout flares, associated with hyperuricemia and crystal deposition, effectively and promptly may reduce the likelihood of developing chronic gout and associated joint destruction, and associated comorbidities. [2] As such, preventive measures for reducing the occurrence of gout flares should be considered.

Our findings with respect to pain and gout flare-related outcomes align with an earlier Cochrane review reported by Sivera et al. [12] However, Sivera et al. included only four RCTs and did not report on any studies involving anakinra. [12] Our results on canakinumab mostly align with this Cochrane review and another systematic review investigating therapies for acute gout overall. [12, 34] Conversely, Sivera et al. reported that rilonacept might not provide superior pain relief than a comparator. [12] This finding contrasts our review, predominantly owing to the additional rilonacept RCTs being included in our review, which reported positive benefits and included other comparators. Arnold et al. conducted a systematic literature review on the safety and efficacy of IL-1-targeted biologics in treating various immune-mediated disorders, including gout, however, their results were limited to RCTs, [11] whereas this review specifically focuses on available evidence on gout.

Overall, our review indicates that IL-1β inhibitors have a good safety profile. AE incidence in canakinumab, anakinra, and rilonacept studies was mostly similar between the IL-1β inhibitor and comparator arms, and most AEs were mild or moderate in severity. SAE incidence in the canakinumab and rilonacept RCTs was similar between the IL-1β inhibitor and comparator arms, although one anakinra study reported that SAE incidence was more frequent in the anakinra arm (1.9%‒7.3%) than the comparator arm (0.0%). [18] Notably, the incidence of infections, which is a risk when taking extended courses of IL-1β inhibitors [9], was generally similar between the IL-1β inhibitor and comparator arms.

IL-1β is a key mediator that drives inflammation in gout, with the role of nod-like receptor pyrin domain containing 3 inflammasome activation being well-established during gout flares. [6, 31, 35] Our results support the efficacy and safety of anti-IL-1β strategies as potential adjuncts to traditional first-line therapies for gout flares, or in patients who are non-responsive or have contraindications to first-line therapies. The cost implications of these therapies should also be considered and detailed in future RCTs, along with efficacy and safety data. [31, 36] Studies assessing the effects of gevokizumab would also be desirable and might give patients and physicians an additional therapeutic option. [31].

## Limitations

A limitation of this review is that only studies published in 2011 or later were eligible for inclusion. Several potentially eligible studies using IL-1β inhibitors for treating gout flares published prior to 2011 were therefore excluded which may have added additional evidence to this review. [37–40] Furthermore, a post-hoc study assessing previously-reported canakinumab trials was excluded, and might have provided further insights into canakinumab’s efficacy and safety. [41] This review is reported as a narrative synthesis which may have a degree of subjectivity and includes some retrospective and post-hoc studies which may introduce a degree of bias in the results of these studies.

## Conclusion

This systematic review demonstrates that canakinumab and rilonacept may be effective for the management of pain associated with gout flares and reducing the frequency of flares, compared to their respective comparators. Anakinra appears not inferior to active comparators, potentially due to the mechanism of action compared to canakinumab and rilonacept, the limited number of trials, and the differences in study design of available trials. More large, well-designed RCTs comparing IL-1β inhibitors with active comparators, with particular focus on safety, real-world evidence, and long-term follow-up, would be warranted to strengthen the evidence base for this therapeutic class. Nevertheless, this systematic literature review suggests that IL-1β-targeted therapy may be beneficial in patients with gout who are unsuitable for current standard therapies.

## Supplementary Information

**Additional file 1.**

## Data Availability

There are no data, per se, associated with this systematic review. All information extracted and included in the review was identified from published articles. All extracted data are therefore included in the main manuscript and Supplemental appendix. Further information can be requested from the authors of the individual included studies.
