# Supplementary material for: Interleukin-1β inhibitors for the management of acute gout flares: a systematic literature review
Source: Arthritis Res Ther. 2023 Jul 25;25:128. doi: 10.1186/s13075-023-03098-4 (PMC10367374; doi:10.1186/s13075-023-03098-4)
Supplement: Supplementary file 1 — Additional file 1. [file 13075_2023_3098_MOESM1_ESM.docx]

# Supplementary Appendix

The authors have provided this appendix to give readers additional information about their work.

## Title

Interleukin-1β inhibitors for the management of acute gout flares: A systematic literature review

## Authors

Naomi Schlesinger, Michael H. Pillinger, Lee S. Simon, Peter E. Lipsky

## Table of contents

| **Supplementary item** | **Page number** |
| --- | --- |
| Supplementary Methods | 2 |
| Supplementary Tables | 6 |
| Supplementary Table S1 | 6 |
| Supplementary Table S2 | 8 |
| Supplementary Table S3 | 9 |
| Supplementary Table S4 | 10 |
| Supplementary Table S5 | 12 |
| Supplementary Table S6 | 16 |
| Supplementary Table S7 | 18 |

# Supplementary Methods

## Search strategy

The following sequence of terms were used and were adapted for each electronic database and the searches were restricted to the years 2011‒2022: ‘gout’ OR ‘acute gout’ OR ‘gouty arthritis’ OR ‘gouty inflammation’ OR ‘gout flares’ OR ‘hyperuricemia’ AND ‘interleukin-1 beta’ OR ‘interleukin-1β’ OR ‘interleukin 1 beta’ OR ‘interleukin-1b’ OR ‘interleukin 1b’ OR ‘interleukin-1 receptor’ OR ‘interleukin 1 receptor’ OR ‘il-1 beta’ OR ‘il 1 beta’ OR ‘il-1b’ OR ‘il 1b’ OR ‘il 1 receptor’ OR ‘il1 receptor’ OR ‘antagon*’ OR ‘inhibit*’ OR ‘block*’ OR ‘antibod*’ OR ‘recomb*’ OR ‘canakinumab’ OR ‘acz885’ OR ‘acz 885’ OR ‘ilaris’ OR ‘gevokizumab’ OR ‘anakinra’ OR ‘antril’ OR ‘kineret’ OR ‘rilonacept’ OR ‘arcalyst’ OR ‘NSAIDS’ OR ‘non-steroidal anti-inflammatory drugs’ OR ‘colchicine’ OR ‘corticosteroids’ OR ‘probalan’ OR ‘indomethacin’ OR ‘lesinurad’ OR ‘zurampic’ OR ‘naproxen’.

An example of the search string that was implemented in the PubMed/MEDLINE database search is detailed below:

1. Gout
2. Acute gout
3. Gouty arthritis
4. Gouty inflammation
5. Gout flares
6. Hyperuricemia
7. #1 OR #2 OR #3 OR #4 OR #5 OR #6
8. interleukin-1 beta
9. interleukin-1β
10. interleukin 1 beta
11. interleukin-1b
12. interleukin 1b
13. interleukin-1 receptor
14. interleukin 1 receptor
15. il-1 beta
16. il 1 beta
17. il-1b
18. il 1b
19. il 1 receptor
20. il1 receptor
21. antagon*
22. inhibit*
23. block*
24. antibod*
25. recomb*
26. canakinumab
27. acz885
28. acz 885
29. Ilaris
30. Gevokizumab
31. Anakinra
32. Antril
33. Kineret
34. rilonacept
35. arcalyst
36. NSAIDS
37. non-steroidal anti-inflammatory drugs
38. colchicine
39. corticosteroids
40. probalan
41. indomethacin
42. lesinurad
43. zurampic
44. naproxen
45. #8 OR #9 OR #10 OR #11 OR #12 OR #13 OR #14 OR #15 OR #16 OR #17 OR #18 OR #19 OR #20 OR #21 OR #22 OR #23 OR #24 OR #25 OR #26 OR #27 OR #28 OR #29 OR #30 OR #31 OR #32 OR #33 OR #34 OR #35 OR #36 OR #37 OR #38 OR #39 OR #40 OR #41 OR #42 OR #43 OR #44
46. #7 AND #45

## Risk of bias in individual studies

The five domains that are covered in the Cochrane Risk of Bias Tool 2 include (i) bias arising from the randomisation process, (ii) bias due to deviations from intended interventions, (iii) bias due to missing outcome data, (iv) bias in the measurement of the outcome, and (v) bias in the selection of the reported results. [14] Signalling questions used to elicit information that was relevant to the assessment of the risk of bias and included the following options: (1) Yes; (2) Probably yes; (3) Probably no; (4) No; and (5) No information. Following the signalling questions, one of the following were assigned to each risk of bias domain: (1) Low risk of bias, (2) Some concerns for risk of bias, or (3) High risk of bias. This process was repeated for each included study. Each study was summarised based on each domain, and details, if applicable, were reported for each study. If answers were disagreed between reviewers, it was discussed between the two reviewers, and if the disagreement could not be resolved, a third independent reviewer decided on the outcome.

For the Downs and Black risk of bias assessment, [15] the number of ‘yes’ scores for each question in the tool was summed, with a maximum total score of 28. Question 27 was modified to ‘Did the study provide a power calculation?’ (yes/no) to be more applicable to the included studies. Conflicts between reviewers were first discussed between the two reviewers, and if a consensus could not be reached, it was resolved by a third reviewer. The questions used in the Downs and Black assessment are detailed in **Supplementary Table S1**.

# Supplementary Tables

**Supplementary Table S1** Downs and Black risk of bias assessment questions

| Question number | Question |
| --- | --- |
| Domain 1: Reporting | |
| *Q1* | Is the hypothesis/aim/objective of the study clearly described? |
| *Q2* | Are the main outcomes to be measured clearly described in the Introduction or Methods section? |
| *Q3* | Are the characteristics of the patients included in the study clearly described? |
| *Q4* | Are the interventions of interest clearly described? |
| *Q5* | Are the distributions of principal confounders in each group of subjects to be compared clearly described? |
| *Q6* | Are the main findings of the study clearly described? |
| *Q7* | Does the study provide estimates of the random variability in the data for the main outcomes? |
| *Q8* | Have all important adverse events that may be a consequence of the intervention been reported? |
| *Q9* | Have the characteristics of patients lost to follow-up been described? |
| *Q10* | Have actual probability values been reported (e.g., 0.035 rather than <0.05) for the main outcomes except where the probability value is less than 0.001? |
| Domain 2: External validity | |
| *Q11* | Were the subjects asked to participate in the study representative of the entire population from which they were recruited? |
| *Q12* | Were those subjects who were prepared to participate representative of the entire population from which they were recruited? |
| *Q13* | Were the staff, places, and facilities where the patients were treated, representative of the treatment the majority of patients receive? |
| Domain 3: Internal validity ‒ bias | |
| *Q14* | Was an attempt made to blind study subjects to the intervention they have received? |
| *Q15* | Was an attempt made to blind those measuring the main outcomes of the intervention? |
| *Q16* | If any of the results of the study were based on “data dredging,” was this made clear? |
| *Q17* | In trials and cohort studies, do the analyses adjust for different lengths of follow-up of patients, or in case-control studies, is the time period between the intervention and outcome the same for cases and controls? |
| *Q18* | Were the statistical tests used to assess the main outcomes appropriate? |
| *Q19* | Was compliance with the intervention/s reliable? |
| *Q20* | Were the main outcome measures used accurate (valid and reliable)? |
| Domain 4: Internal validity – confounding (selection bias) | |
| *Q21* | Were the patients in different intervention groups (trials and cohort studies) or were the cases and controls (case-control studies) recruited from the same population? |
| *Q22* | Were study subjects in different intervention groups (trials and cohort studies) or were the cases and controls (case-control studies) recruited over the same period of time? |
| *Q23* | Were study subjects randomised to intervention groups? |
| *Q24* | Was the randomised intervention assignment concealed from both patients and health care staff until recruitment was complete and irrevocable? |
| *Q25* | Was there adequate adjustment for confounding in the analyses from which the main findings were drawn? |
| *Q26* | Were losses of patients to follow-up taken into account? |
| Domain 5: Power | |
| *Q27* | Did the study provide a power calculation? (Question modified* from the original Downs and Black tool) |
| Questions are derived from Downs and Black, 1998. [15] *Original Q27: Did the study have sufficient power to detect a clinically important effect where the probability value for a difference being due to chance is less than 5%? | |

**Supplementary Table S2** Intervention details of the non-RCTs

| Author | Study design | Number of patients by treatment group | Intervention and comparator | Primary endpoint |
| --- | --- | --- | --- | --- |
| ANK (*N*=3) | | | | |
| Ghosh et al, 2013 [26] | Retrospective chart review | 26 hospitalised patients treated with ANK for 40 GFs  Single dose: *n*=4  q.d. for 2 d: *n*=4  q.d. for 3 d: *n*=17  q.d. for 4 d: *n*=1  q.d. for 5 d: *n*=3  One dose every other day for 3 d: *n*=7  b.i.d. for 5 d: *n*=4 | **Intervention:** ANK doses (single dose, q.d. for 2 d, q.d. for 3 d, q.d. for 4 d, q.d. for 5 d, one dose every other day for 3 d, b.i.d. for 5 d)  **Comparator:** None | NS |
| Liew & Gardener, 2019 [27] | Retrospective observational study | 100 patients hospitalised at 2 hospitals (93 had gout)  100 mg, once: *n*=24  100 mg q.d.: 2 doses: *n*=13, 3 doses: *n*=52, >3 doses: *n*=8  100 mg, every other day: 2 doses: *n*=4, 3 doses: *n*=13, >3 doses: *n*=1 | **Intervention:** ANK doses (100 mg once, 100 mg q.d. [2 doses/3 doses/>3 doses], 100 mg every other day [2 doses/3 doses/>3 doses])  **Comparator:** None | NS |
| Ottaviani et al, 2013 [28] | Retrospective chart review | 40 patients | **Intervention:** 100 mg ANK, q.d. for 3 d, 100 mg ANK q.d. (<15 d), 100 mg ANK every 2 d (<15 d), 100 mg ANK q.d. (>15 d, followed by dose spacing)  **Comparator:** None | NS |
| CAN (*N*=1) | | | | |
| Solomon et al 2018 [29] | Post hoc analysis of data from CANTOS, a PBO-controlled trial with a parallel design | PBO: *n*=3343 (250 had a history of gout)  CAN: *n*=6716 (512 had a history of gout) | **Intervention:** 3 dosages of CAN (50, 150, and 300 mg) every 3 months  **Comparator:** PBO | GFs were assessed in a blinded fashion, but they were not prespecified as an outcome. CV endpoints were the primary endpoints |
| ANK, anakinra; b.i.d., twice daily; CAN, canakinumab; CV, cardiovascular; d, day(s); GF, gout flare; n, number of patients in group; N, number of studies; NS, not specified; q.d., once daily; PBO, placebo; RCT, randomised controlled trial. | | | | |

**Supplementary Table S3** Baseline demographic and disease characteristics of the non-RCTs

| Author | Sex, male (%) | Age range, years, mean (SD) | Disease duration, years, mean (SD) | Number of GFs, mean (SD) | Presence of tophi, % | Reason for prescribing IL-1β inhibitors |
| --- | --- | --- | --- | --- | --- | --- |
| ANK (*N*=3) | | | | | | |
| Ghosh et al, 2013 [26] | 84.6% | 56.8 (range: 32‒86) | NR | NR | 40.9% | Patients who were resistant to standard therapy or had significant comorbidities that precluded the use of NSAIDs/CLC/steroids |
| Liew & Gardener, 2019 [27] | 82.0% | 60.0 (15.0) | NR | NR | NR | Comorbidities (%): 84%  Failure of other therapies (%): 48%  Reason not documented (%): 8% |
| Ottaviani et al 2013 [28] | 80.0% | 60.0 (13.9) | 8.7 (8.7) | NR | 79% | **Reason for ANK use (%):**  Non-response to conventional therapies:  CLC: 60.0%  NSAIDs: 27.5%  Steroids: 17.5%  AEs/contraindication to conventional therapies:  CLC: 40.0%  NSAIDs: 72.5%  Steroids: 22.5% |
| CAN (*N*=1) | | | | | | |
| Solomon et al 2018 [29] | PBO: 74.1%  CAN: 74.4% | **Median (IQR):**  PBO: 61 (54‒68)  CAN: 61 (54‒68) | NR | NR | NR | NR |
| AE, adverse event; ANK, anakinra; CAN, canakinumab; CLC, colchicine; GF, gout flare; IL-1β, interleukin-1 beta; N, number of studies; NR, not reported; NSAID, non-steroidal anti-inflammatory drug; PBO, placebo; RCT, randomised controlled trial; SD, standard deviation. | | | | | | |

**Supplementary Table S4** Efficacy results of the non-RCTs

| Author | Number of GFs | Severity of GFs or pain | Duration of GFs and/or time between GFs | Synovitis | Other outcomes |
| --- | --- | --- | --- | --- | --- |
| ANK (*N*=3) | | | | | |
| Ghosh et al, 2013 [26] | NR | 67% of ANK courses resulted in significant pain improvement within 24 h; 18% of courses resulted in significant pain improvement within 48 h. 85% of patients had significant pain improvement within 2 d. 15% required 3–6 d to achieve pain improvement | NR | Complete resolution of symptoms (pain, swelling, erythema and warmth) was achieved in 72.5% of patients within 5 d, and by 10 d, all but 1 patient had complete resolution of symptoms | NR |
| Liew & Gardener, 2019 [27] | NR | Significant response or complete resolution by 4 d: *n*=86 (74.8% of episodes)  Delayed response >4 d: *n*=2 (1.7% of episodes)  Partial response: *n*=7 (6.1% of episodes)  Nonresponse: *n*=6 (5.2% of episodes)  Insufficient information: *n*=14 (12.2% of episodes)  66 (57.4%) episodes had partial or complete response within 1 d of the first dose | NR | NR | NR |
| Ottaviani et al 2013 [28] | NR | **Response to ANK:**  Good: *n*=36 (90%)  Partial: *n*=2 (5%)  None: *n*=2 (5%)  **VAS change:**  73.5 (70.0‒80.0) to 25.0 (20.0‒32.5) mm) | After a median FU (IQR) of 7.0 (2.0‒13.0) months, relapse occurred in 13 (32.5%) patients: median (IQR) delay: 15.0 d (10.0‒70.0) | NR | Some patients took CLC, NSAIDs, steroids; relapse occurred particularly in patients not receiving therapy to prevent GFs (7/10 vs 6/30). No relapse occurred with long-term use of ANK (>15 d) |
| CAN (*N*=1) | | | | | |
| Solomon et al 2018 [29] | 195 patients had ≥1 GF; 45% (*n*=87) had a history of gout and 17% (*n*=34) had repeated GFs.  PBO: 99 GFs (IR: 0.80 GFs per 100 pt-y)  CAN group: 96 GFs (IR: 0.38 GFs per 100 pt-y) | Risk for first GF during FU was reduced by 52% (HR: 0.48 [CI: 0.36‒0.63]) in patients receiving CAN vs PBO | Median time between treatment and GF: 74 d (IQR: 28‒145 d) | NR | NR |
| ANK, anakinra; CAN, canakinumab; CI, confidence interval; CLC, colchicine; d, day(s); FU, follow up; GF, gout flare; h, hour/s; HR, hazard ratio; IQR, interquartile range; IR, incidence rate; n, number of patients in group; N, number of studies; NR, not reported; NSAID, non-steroidal anti-inflammatory drug; PBO, placebo; pt-y, patient-years; RCT, randomised controlled trial; VAS, visual analogue scale. | | | | | |

**Supplementary Table S5** Safety results of the RCTs and non-RCTs

| Author | Number and types of AEs (%) | Number and types of SAEs (%) | Dropouts/discontinuations/deaths due to AEs |
| --- | --- | --- | --- |
| RCTs (*N*=10) | | | |
| CAN (*N*=3) | | | |
| Schlesinger et al 2011a [20] | **Any AEs:**  CAN: 41.3%  TA: 42.1%  All AEs except 2 were mild/moderate | CAN: 0.0%  TA: 0.0% | No patients discontinued due to AEs and 0 deaths were reported |
| Schlesinger et al, 2011b [21] | **AEs:**  CAN 25 mg: 52.7%  CAN 50 mg: 55.6%  CAN 100 mg: 51.9%  CAN 200 mg: 51.9%  CAN 300 mg: 54.7%  CAN q4wk: 58.5%  CAN any dose: 54.2%  CLC 0.5 mg: 53.7%  Most AEs were mild or moderate in severity, and there was no evidence of a dose-response for any AE  **Most common AEs:**  CAN 25 mg: HTN (10.9%)  CAN 50 mg: Arthralgia (9.3%)  CAN 100 mg: Arthralgia (7.4%)  CAN 200 mg: HTN (9.3%)  CAN 300 mg: Headache (11.3)  CAN q4wk: Headache (5.7%) + URTI (5.7%) + nasopharyngitis (5.7%) + rash (5.7%)  CLC 0.5 mg: Headache (5.6%) | **SAEs:**  CAN 25 mg: 3.6%  CAN 50 mg: 3.7%  CAN 100 mg: 5.6%  CAN 200 mg: 5.6%  CAN 300 mg: 5.7%  CAN q4wk: 1.9%  CAN any dose: 4.3%  CLC 0.5 mg: 5.6%  2 SAEs were possibly related to study medication (1, CAN 25 mg; 1, CLC 0.5 mg) | **SAEs leading to discontinuation (%):**  Any CAN: 1.2%  CLC: 1.9%  1 death (CLC group) due to a possible MI (not related to study medication) |
| Schlesinger et al, 2012 [19] | **AEs for pooled patients (over 24 wk):**  CAN: 66.2%  TA: 52.8%  **Most common AEs:**  CAN: Back pain (5.8%)  TA: HTN (5.7%) | **Pooled patients (over 24 wk):**  CAN: 7.6%  TA: 3.1% | There were 2 deaths, neither considered related to the study medication (1, TA group [pulmonary embolism]; 1, CAN group [intracranial haemorrhage]) |
| ANK (*N*=2) | | | |
| Janssen et al, 2019 [16] | **Any AEs:**  TaU: 46.7%  ANK: 34.9%  **Most common AEs:**  TaU: Diarrhoea (18.4%) and other AE (20.4%)  ANK: MSK pain (16.2%) and other AE (24.3%) | 0 SAEs were reported | 0 deaths were reported |
| Saag et al, 2021 [18] | **All TEAEs:**  TA 40 mg: 40.7%  ANK 100 mg: 38.2%  ANK 200 mg: 55.8%  **Most common AEs:**  Hypertriglyceridemia (5 patients), neutropenia (4 patients), & several types of injection site reactions (erythema, pruritus, or swelling) were the most frequently reported AEs in the ANK groups. Headache (2 patients) was most common in the TA group. Most AEs were mild | **All SAEs:**  TA 40 mg: 0.0%  ANK 100 mg: 7.3%  ANK 200 mg: 1.9% | **Discontinuations:**  TA 40 mg: 5.6%  ANK 100 mg: 1.8%  ANK 200 mg: 3.8%  0 deaths were reported |
| RL (*N*=5) | | | |
| Mitha et al, 2013 [17] | **Any AEs:**  PBO: 61.0%  RL 80 mg: 68.3%  RL 160 mg: 61.9%  **TEAEs:**  PBO: 8.5%  RL 80 mg: 31.7%  RL 160 mg: 25.0%  **Most common AEs:**  PBO: URTI (12.2%)  RL 80 mg: Injection site reactions (12.2%) and URTI (12.2%)  RL 160 mg: Injection site reactions (17.9%) | **Any SAEs:**  PBO: 4.9%  RL 80 mg: 6.1%  RL 160 mg: 3.6% | **Discontinuations (%):**  PBO: 0.0%  RL 80 mg: 3.7%  RL 160 mg: 0.0%  0 deaths were reported |
| Schumacher et al, 2012a [23] | **Any TEAE:**  PBO: 59.5%  RL: 61.0%  **Most common AEs:**  PBO: URTI (9.5%) and joint-related signs and symptoms (9.5%)  RL: URTI (9.8%) and injection and infusion site reactions (9.8%) | **Any SAE:**  PBO: 4.8%  RL: 2.4% | **Discontinuations (%):**  PBO: 7.1%  RL: 2.4%  0 deaths were reported |
| Schumacher et al, 2012b [22] | **Any AEs:**  PBO: 60.8%  RL 80 mg: 61.3%  RL 160 mg: 65.4%  **TEAEs:**  PBO: 7.6%  RL 80 mg: 17.5%  RL 160 mg: 32.1%  **Most common AEs:**  PBO: Pain in extremity (5.1%)  RL 80 mg: Injection site reactions (8.8%)  RL 160 mg: Injection site reactions (19.8%) | **Any SAEs:**  PBO: 3.8%  RL 80 mg: 3.8%  RL 160 mg: 3.7% | **Discontinuations:**  PBO: 5.1%  RL 80 mg: 5.0%  RL 160 mg: 3.7%  0 deaths were reported |
| Sundy et al, 2014 [24] | **Primary outcome:**  **Any AEs:**  PBO: 59.1%  RL: 66.6%  **TEAEs:**  PBO: 13.0%  RL: 27.5%  **Most common AEs:**  PBO: URTI (10.3%)  RL: Injection site reactions (15.2%) | **All SAEs:**  PBO: 3.9%  RL: 3.1%  **TESAEs:**  PBO: 0.6%  RL: 0.1% | **Discontinuations (%):**  PBO: 3.0%  RL: 4.7%  **Deaths (%):**  PBO: 0.9%  RL: 0.3%  1 death (PBO) was related to study treatment. Other causes of death: 2 MIs (RL), 1 cerebrovascular event (RL), 1 sudden cardiac death (PBO), 1 collapsed lung (PBO) |
| Terkeltaub et al, 2013 [25] | **Any AE:**  PBO+IND: 29.9%  RL+IND: 46.6%  RL+PBO: 36.0%  **Most common AEs:**  PBO+IND: Headache (7.8%), dizziness (5.2%)  RL+IND: Headache (5.5%), dizziness (4.1%)  RL+PBO: Headache (9.3%), dizziness (2.7%) | **Any SAE:**  PBO+IND: 0.0%  RL+IND: 4.1%  RL+PBO: 0.0% | **Discontinuation (%):**  PBO+IND: 2.6%  RL+IND: 1.4%  RL+PBO: 1.3%  1 death was due to hypertensive cardiomyopathy that was not related to RL treatment |
| Non-RCTs (*N*=4) | | | |
| ANK (*N*=3) | | | |
| Ghosh et al, 2013 [26] | ANK was well tolerated, with no injection site reactions or allergic reactions noted. There were no instances of documented leukopenia. Seven patients with perioperative GFs and 4 patients who were immunosuppressed were given ANK without AEs. Two patients under appropriate antibiotic treatment for infection were given ANK without exacerbation of infection. One patient developed a postoperative wound infection; however, his wound site was draining and possibly infected 4 d prior to the first ANK dose | NR | NR |
| Liew & Gardener, 2019 [27] | Overall, ANK was well tolerated. Two patients had leukopenia attributed to ANK (1 new, 1 with worsening of pre-existing white blood cell counts). Worsening of bicytopenia, injection site reaction, and nausea occurred in 1 patient each | NR | NR |
| Ottaviani et al, 2013 [28] | No patient reported ANK-related skin hypersensitivity. Seven infectious complications (mostly staphylococcal) were reported in 6 patients. Of the 6 patients, 5 restarted ANK after infection resolution. | NR | NR |
| CAN (*N*=1) | | | |
| Solomon et al 2018 [29] | NR | NR | NR |
| ADA, antidrug antibody; AE, adverse event; ANK, anakinra; CAN, canakinumab; CLC, colchicine; d, day(s); GF, gout flare; HTN, hypertension; IND, indomethacin; MI, myocardial infarction; MSK, musculoskeletal; N, number of studies; NR, not reported; q4wk, every 4 weeks; PBO, placebo; RCT, randomised controlled trial; RL, rilonacept; SAE, serious adverse event; TA, triamcinolone acetonide; TaU, treatment as usual; TEAE, treatment emergent adverse event; TESAE, treatment-emergent serious adverse event; URTI, upper respiratory tract infection; wk, week(s). | | | |

**Supplementary Table S6** Additional efficacy results of the RCTs and non-RCTs

| Author | Quality of life | Biomarkers | Global response to treatment |
| --- | --- | --- | --- |
| RCTs (*N*=10) | | | |
| CAN (*N*=3) | | | |
| Schlesinger et al, 2011a [20] | **SF-36 PCS:**  At 7 d, all SF-36 PCS aspects improved and was greatest in CAN 150 mg (+12.0 [10.0]; 7 d post dose: 48.3 [8.6]) and exceeded that of the US general population by 8 wk (52.8 [6.7]). In TA at 7 d, SF-36 aspects improved (+8.5 [10.4]; 7 d post dose: 41.9 [9.5]), and by 8 wk, it was 47.1 (11.2). In CAN 150 mg group, the greatest improvement was the physical functioning and bodily pain domains | **CRP:**  At 7 d, CRP levels were normalised (≤3.0 mg/L) in 46.4%‒72.4% of CAN patients vs 41.1% of TA patients. In CAN 150 mg, CRP normalisation was significantly greater than the TA group at 7 d, 4 wk, and 8 wk post-dose | At 72 h, CAN 150 mg was significant for treatment response via patient global self-assessment and PGA vs TA patients |
| Schlesinger et al 2011b [21] | NR | **CRP:**  An initial decrease in median CRP levels was seen in all treatment groups. For all CAN doses ≥50 mg, median CRP values remained consistently lower than the CLC group throughout the study | NR |
| Schlesinger et al, 2012 [19] | NR | **CRP pooled patients (72 h post dose; median [IQR]):**  CAN: 4.4 (1.8‒9.8)  TA: 6.1 (2.2‒17.6)  CRP levels were consistently suppressed over 24 wk and were lower at each time point in the CAN group | **Pooled patients (OR [95% CI] 72 h post-dose):**  **Patient global self-assessment:**  CAN: 2.2 (1.6‒3.1)  **PGA:**  CAN: 2.3 (1.6‒3.3) |
| ANK (*N*=2) | | | |
| Janssen et al 2019 [16] | NR | **CRP after 7 d (mean [SE] reduction):**  TaU: 13.9 (7.1)  ANK: 11.1 (7.5) | **PGA (mean [SE]:**  TaU: 4.4 (0.3) to 6.7 (0.3)  ANK: 4.9 (0.4) to 7.3 (0.3) |
| Saag et al, 2021 [18] | NR | **CRP:**  ANK patients had significantly reduced CRP levels at 72 h and on day 8, vs TA (MD: ‒0.9 [95% CI: ‒1.6 to ‒0.3] and ‒0.6 [95% CI: ‒1.1 to ‒0.0]). On day 15, CRP levels were significantly reduced in the TA group vs the total ANK group (MD: 0.8 [95% CI: 0.2‒1.4]) | Mean patient assessment of global response to treatment was significantly better in the total ANK group vs TA group on day 8 (−0.6 [95% CI: ‒1.0 to ‒0.2]) and day 15 (−0.4 [95% CI: ‒0.9 to ‒0.0]).  **PGA:**  The mean PGA of treatment response was significantly better in ANK patients on day 8 (−0.4 [95% CI: ‒0.8 to 0.0]) |
| RL (*N*=5) | | | |
| Mitha et al, 2013 [17] | NR | NR | NR |
| Schumacher et al, 2012a [23] | NR | NR | NR |
| Schumacher et al, 2012b [22] | NR | NR | NR |
| Sundy et al, 2014 [24] | NR | Treatment with RL was associated with small mean increases in ALT, AST, TGs and CPK. There were small mean decreases with RL in neutrophil and platelet counts | NR |
| Terkeltaub et al, 2013 [25] | NR | **CRP:**  All treatment groups were characterised by a general reduction from baseline in hsCRP from initiation of treatment to the safety FU at day 31. On day 4, these reductions were significantly greater with RL+IND and RL relative to IND | NR |
| Non-RCTs (*N*=4) | | | |
| ANK (*N*=3) | | | |
| Ghosh et al, 2013 [26] | NR | NR | NR |
| Liew & Gardener, 2019 [27] | NR | NR | NR |
| Ottaviani et al, 2013 [28] | NR | **CRP:**  CRP reduced from 130.5 (55.8‒238.8) to 16.0 (5.0‒ 29.5) mg/L | NR |
| CAN (*N*=1) | | | |
| Solomon et al 2018 [29] | NR | **CRP:**  hsCRP levels decreased in all patients receiving CAN. Reductions were largest among those receiving higher doses. | NR |
| ALT, alanine transferase; ANK, anakinra; AST, aspartate transferase; CAN, canakinumab; CI, confidence interval; CLC, colchicine; CPK, creatine phosphokinase; CRP, C-reactive protein; d, day(s); FU, follow-up; h, hour(s); hsCRP, high sensitivity C-reactive protein; IQR, interquartile range; IND, indomethacin; MD, mean difference; N, number of studies; NR, not reported; OR, odds ratio; PCS, physical component summary; PGA, physician’s global assessment; RCT, randomised controlled trial; RL, rilonacept; SE, standard error; SF-36, short-form 36; TA, triamcinolone acetonide; TaU, treatment as usual; TGs, triglycerides; US, United States; wk, week(s). | | | |

**Supplementary Table S7** A summary of the risk of bias assessment for non-RCTs using the Downs and Black assessment tool

| **Question** | **Ghosh et al 2013 [26]** | **Liew & Gardener 2019 [27]** | **Ottaviani et al 2013 [28]** | **Soloman et al 2018 [29]** |
| --- | --- | --- | --- | --- |
| ***Domain 1: Reporting*** | | | | |
| *Q1* | N | Y | Y | Y |
| *Q2* | N | Y | Y | Y |
| *Q3* | N | Y | Y | Y |
| *Q4* | Y | Y | Y | Y |
| *Q5** | N | N | Y | Y |
| *Q6* | Y | Y | Y | Y |
| *Q7* | N | Y | Y | Y |
| *Q8* | Y | Y | Y | N |
| *Q9* | N | N | N | N |
| *Q10* | N | N | Y | Y |
| ***Domain 2: External validity*** | | | | |
| *Q11* | UD | UD | UD | Y |
| *Q12* | UD | UD | UD | Y |
| *Q13* | UD | UD | UD | UD |
| ***Domain 3: Internal validity ‒ bias*** | | | | |
| *Q14* | N | N | N | Y |
| *Q15* | N | N | N | Y |
| *Q16* | Y | Y | Y | Y |
| *Q17* | N | N | N | Y |
| *Q18* | N | Y | Y | Y |
| *Q19* | Y | Y | Y | Y |
| *Q20* | N | Y | Y | Y |
| ***Domain 4: Internal validity ‒ confounding (selection bias)*** | | | | |
| *Q21* | UD | N | N | Y |
| *Q22* | N | N | UD | Y |
| *Q23* | N | N | N | Y |
| *Q24* | N | N | N | Y |
| *Q25* | N | N | N | Y |
| *Q26* | N | N | N | Y |
| ***Domain 5: Power*** | | | | |
| *Q27* | N | N | N | N |
| **Sum of ‘yes’ scores (of 28)** | **5** | **11** | **14** | **24** |
| A greater number of ‘yes’ scores indicates a lower degree of bias.  *All questions except for Q5 give one point towards the total; Q5 gives 2 points for ‘yes’ and 1 point for ‘partially’.  ‘Unable to determine’ indicates that either the data could not be found in the study, or the question is not applicable to the study.  N, no; Q, question; RCT, randomised controlled trial; Y, yes; UD, unable to determine. | | | | |
